# Stop Eating and Start Moving: One Switch Does Both

**DOI:** 10.1371/journal.pbio.1001894

**Published:** 2014-06-24

**Authors:** Richard Robinson

**Affiliations:** Freelance Science Writer, Sherborn, Massachusetts, United States of America

At its most elemental level, a behavior is a series of coordinated muscle contractions that accomplishes some goal. Those contractions are driven by motor neurons, whose firing is controlled by “central pattern generators,” suites of central nervous system neurons functionally linked to generate simple motor outputs, such as chewing, swallowing, or breathing.

Switching between behaviors requires suppression of one set of patterns and activation of another. The larva of the fruit fly, for instance, doesn't feed while it is in motion, and vice versa, and so must stop doing the one in order to engage in the other. How it does so, and more broadly, how organisms coordinate switching between motor patterns, is not well understood. In this issue of *PLOS Biology*, Andreas Schoofs, Michael J. Pankratz, and colleagues show that a single cluster of neurons in the fly central nervous system simultaneously suppresses feeding movements and induces food-seeking movements.

Feeding behavior in the fly larva includes three motor patterns—head tilting movements, mouth hook movements, and pharyngeal pumping—that together allow the larva to find, ingest, and swallow food, such as yeast cells. The authors previously identified three nerves that together control this set of movements. To discover central neurons that modulate the activity of these nerves, they selectively activated neurons expressing different neurotransmitters or neuropeptides using an inducible genetic tool. By recording the output of the nerves as they turned on one or another gene, they found four classic neurotransmitters and one neuropeptide with significant effects on one or another feeding motor pattern. Acetylcholine, glutamic acid, and serotonin each increased activity for all patterns, while dopamine and the neuropeptide hugin selectively decreased pharyngeal pumping.

**Figure 1:**
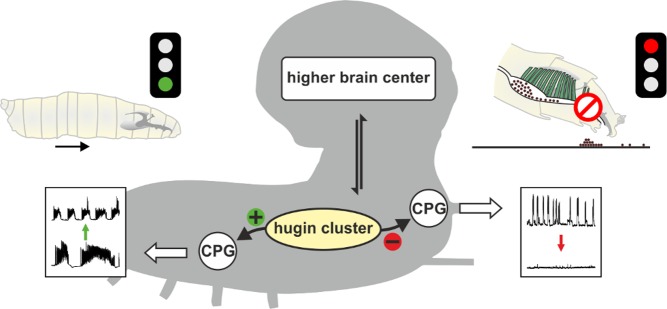
Andreas Schoofs, Michael J. Pankratz, and colleagues showed that activation of a 20-cell neuronal cluster in *Drosophila* larva expressing the neuropeptide hugin simultaneously stops food intake and induces locomotion. CPG, central pattern generators.

While dopamine is widely expressed, hugin is limited to 20 neurons tightly clustered together at the base of the brain, just below the fly higher brain centers. Further investigation showed that, in addition to suppressing pharyngeal pumping, activation of hugin neurons induced wandering, a food-seeking behavior, and increased the frequency of contraction of abdominal muscles, which propel the larva through the medium (see Figure 1). When hugin expression was suppressed but the neurons were stimulated to fire nonetheless, pharyngeal pumping remained unsuppressed and wandering induction uninduced, indicating their reliance on hugin. Abdominal muscle contractions, however, were still increased, suggesting that hugin neurons also release another, as-yet-unidentified neurotransmitter.

Previous anatomic work had shown that of the 20 hugin neurons, four extend processes down the length of the nerve cord, suggesting they might play a role in controlling abdominal muscles. By selectively activating or ablating these cells or the 16 others in the cluster, the authors showed that these four indeed increased abdominal muscle contraction frequency, without hugin release. The other 16, in turn, whose targets include the pharynx, were shown to be responsible for suppressing feeding and induction of wandering in a hugin-dependent fashion.

While increasing hugin release could suppress feeding and induce locomotion, the opposite was not true—reducing hugin release did not increase feeding or suppress locomotion, indicating that, like the brake and accelerator of a car, each of these actions is under the control of two independent regulatory systems exerting opposite effects.

Further work will be needed to understand these other systems and to determine how the hugin-dependent central pattern generator is itself regulated by higher centers. But the authors made important headway here, showing that a subset of the hugin neurons connect to the protocerebrum (part of the fly's brain), and that the protocerebrum contributes to control of the hugin neurons. While working out all the mutually interacting systems that contribute to even such simple behaviors remains a major challenge, these new discoveries have made the task more tractable.

**Schoofs A, Hückesfeld S, Schlegel P, Miroschnikow A, Peters M, et al. (2014) Selection of Motor Programs for Suppressing Food Intake and Inducing Locomotion in the** ***Drosophila*** **Brain.** doi:10.1371/journal.pbio.1001893

